# Weighted log-linear models for service delivery points in Ethiopia: a case of modern contraceptive users at health facilities

**DOI:** 10.1186/s12978-018-0520-9

**Published:** 2018-05-10

**Authors:** Demeke Lakew Workie, Dereje Tesfaye Zike, Haile Mekonnen Fenta, Mulusew Admasu Mekonnen

**Affiliations:** 1 Statistics Department, Science College, Bahir Dar University, Bahir Dar, Ethiopia; 2 PMA2020/Ethiopia project & John Snow Inc (JSI) SEUHP/Ethiopia project, http://www.pma2020.org/Ethiopia

**Keywords:** Negative binomial, Number of contraceptive users, Service delivery points, Weighted log-linear

## Abstract

**Background:**

Ethiopia is among countries with low contraceptive usage prevalence rate and resulted in high total fertility rate and unwanted pregnancy which intern affects the maternal and child health status. This study aimed to investigate the major factors that affect the number of modern contraceptive users at service delivery point in Ethiopia.

**Methods:**

The Performance Monitoring and Accountability2020/Ethiopia data collected between March and April 2016 at round-4 from 461 eligible service delivery points were in this study. The weighted log-linear negative binomial model applied to analyze the service delivery point’s data.

**Results:**

Fifty percent of service delivery points in Ethiopia given service for 61 modern contraceptive users with the interquartile range of 0.62. The expected log number of modern contraceptive users at rural was 1.05 (95% Wald CI: − 1.42 to − 0.68) lower than the expected log number of modern contraceptive users at urban. In addition, the expected log count of modern contraceptive users at others facility type was 0.58 lower than the expected log count of modern contraceptive users at the health center. The numbers of nurses/midwives were affecting the number of modern contraceptive users. Since, the incidence rate of modern contraceptive users increased by one due to an additional nurse in the delivery point.

**Conclusion:**

Among different factors considered in this study, residence, region, facility type, the number of days per week family planning offered, the number of nurses/midwives and number of medical assistants were to be associated with the number of modern contraceptive users. Thus, the Government of Ethiopia would take immediate steps to address causes of the number of modern contraceptive users in Ethiopia.

## Plain English summary

In Ethiopia, there is a high total fertility rate and unwanted pregnancy due to low contraceptive prevalence rate. This study aimed to investigate the major factors that affect the number of modern contraceptive users at service delivery point in Ethiopia. The weighted log-linear negative binomial model applied to analyze the Performance Monitoring and Accountability2020/Ethiopia data.

The 461 eligible service delivery points were in this study. Fifty percent of service delivery points in Ethiopia given service for 61 modern contraceptive users with the interquartile range of 0.62. Among different factors considered in this study, residence, region, facility type, the number of days per week family planning offered, the number of nurses/midwives and number of medical assistants were found to be associated factors for the number of modern contraceptive users in Ethiopia.

In conclusion, the Government of Ethiopia both regional and federal would take immediate steps to address the causes of the number of modern contraceptive users in Ethiopia.

## Background

Globally, each year, nearly 350,000 women die while another 50 million suffer illness and disability from complications of pregnancy and childbirth [1]. In developing countries, millions of sexually active women aged 15–49 want to avoid pregnancy and delay childbearing for at least 2 years or want to stop pregnancy and limit their family size but have an unmet need for family planning (FP) [2]. About 25% of women who would like to postpone their next birth by 2 years do not currently use a contraceptive method. This need could be met by improving contraceptive knowledge and the supply of reproductive health services so that women can better plan their families [3]. It has been reported that Ethiopia is one among six countries that contribute to about 50% of the maternal deaths along with India, Nigeria, Pakistan, Afghanistan and the Democratic Republic of Congo [1]. The total fertility rate of Ethiopia is 4.6 children per woman, contraceptive prevalence rate (CPR) is only 36% and an unmet need for family planning is 22% for married women [4], 24% of total women age 15–49 years [5] and 16.2% among all women aged 15–49 years [6]. If Ethiopia follows its current rate of growth, its population will double in the next 30 years, hitting 210 million by 2060. For fertilities to fall to those low levels, increases the use of modern contraceptive methods and family planning service delivery points play a significant contribution especially in less developed countries including Ethiopia. At present, contraceptive methods which are free of cost is provided in both governmental and NGO health facilities in Ethiopia at hospitals, clinics, health centers, and health posts [7]. But, Ethiopia is among countries with low contraceptive prevalence rate, with only 36% [4]. This resulted in high total fertility rate and unwanted pregnancy which intern affects the maternal and child health status [8].

Current use of modern contraceptive methods is one of the indicators most frequently used to assess the success of family planning programs. In Ethiopia, the variations of modern contraceptive use observed among regions, place of residence, marital status, wealth index and other factors [4, 5]. This situation indicated that the assumption of conditional independence of responses of individuals on the probability of contraceptive users who are living in the same area (cluster) given the covariates may not be longer valid. This indicates that current contraceptive use may be affected by unobserved regional and clustering effects at the different level of the factors [9]. The modern contraceptive prevalence rate in Ethiopia is varied from 1.4% in Somali to 50.1% in Addis Ababa across regions and 49.8% in Urban to 32.4 in rural via residence [4]. The success of any policy or family planning program intervention depends on a correct understanding of the socioeconomic, geographic, demographic, and behavioral factors which may influence the family planning health facilities and modern contraceptive users. It is believed that population growth and family planning health facilities are closely related concepts. The principal findings and recommendations for strengthening the modern contraceptive users are availability and access to services, health facilities readiness, staffing training and improving the quality of care [10, 11]. Therefore, this study aimed to investigate the major factors that affect the modern contraceptive users at service delivery point in Ethiopia using weighted log-linear negative binomial model.

## Methods

### Data source, sampling design, and sample size

**Fig. 1:**
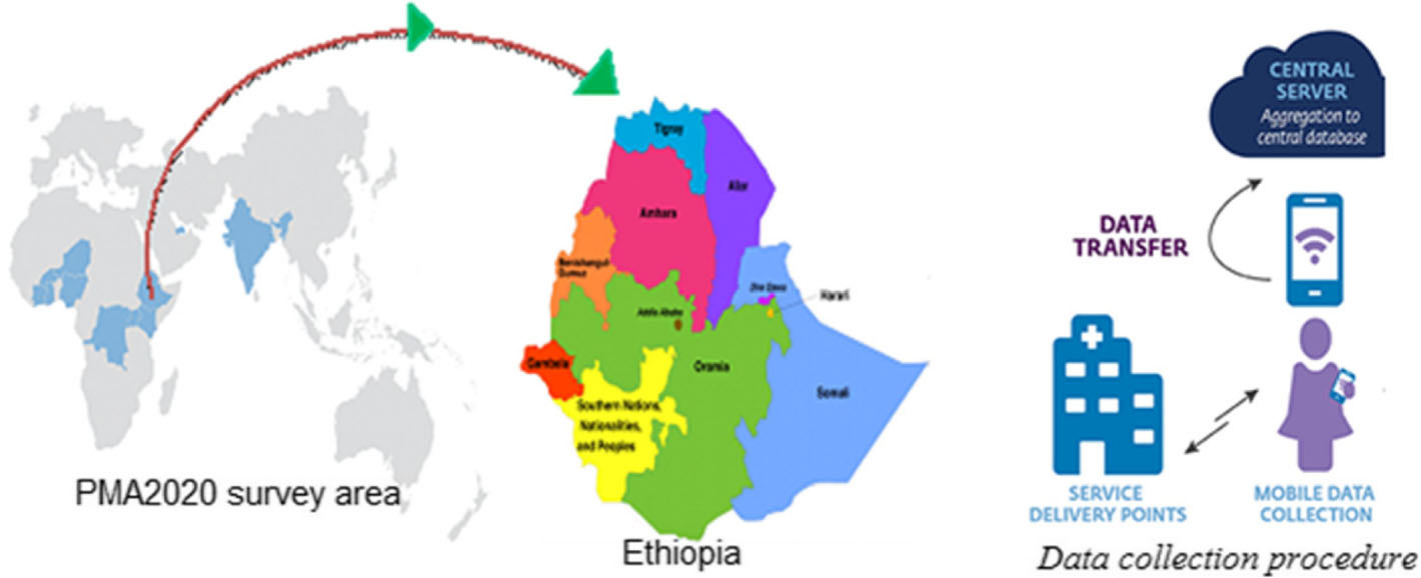
Study area and data collection procedures

The PMA2020/Ethiopia-R4 data was collected by Addis Ababa University’s School of Public Health at the College of Health Sciences (AAU/SPH/CHS), in collaboration with regional universities, the Federal Ministry of Health and the Central Statistics Agency under the aegis of the Bill & Melinda Gates Institute for Population and Reproductive Health at the Johns Hopkins Bloomberg School of Public Health. The PMA2020/Ethiopia project was applied a two-stage stratified sample selection and stratification was achieved by separating each region into urban and rural areas. A sample of 461 eligible service delivery points (SDP) was considered for this study. The data collection was conducted between March and April 2016 by trained women who attained a high school diploma or higher level of education using smartphones. The study area and data collection procedures revealed in Fig. 1 [12].

### Measurements

The response variable for this study was defined as the total number of visitors for modern contraceptive users at service delivery points during the last complete month preceding the survey. The predictor variables that included in this study were Region, residence (rural and urban), type of health facility (Health center and others (include: Health post, Hospital, Clinic and Pharmacy/drug shop/retail), advanced facility (Yes or No), facility supports by CHVs (Yes or No), the number of opening days per a week to offer family planning (5 days or below and above 5 days), total number of doctors, total number of nurses/midwives, total number of health officers and total number of pharmacists [13]. Data were entered into STATA-12 and analyzed using SAS-9.2.

### Statistics analysis

A common model for count data is the Poisson model by assuming that the distribution has mean and variance equally [14]. Often, this does not hold true in real data, the sample variance is considerably larger than mean called over-dispersion and rarely smaller called under-dispersion [15]. An over-dispersed model which assumes equidispersion can result in misleading inferences and conclusions, as over-dispersion can lead to the underestimation of parameter standard errors and falsely increase the significance of beta parameters [16, 17]. Hinde and Demetrio have published the issue of over-dispersion in both binary and count data whereas more recently, Hayat and Higgins have published a review of Poisson regression and over-dispersion [18, 19].

Contraceptive user’s data which is an example of count data, often exhibit larger variance than would be expected from the Poisson assumption [20]. There are a number of strategies for accommodating over-dispersion. One of the approaches among a lot is a model in which μ was a random variable with a gamma distribution leading to a negative binomial distribution (NB) for the count data [20]. NB regression handles dispersion issues by modeling the dispersion parameter of the response variable. The relationship between variance and mean for NB distribution has the form of var. (Y_i_) =${\mu}_i+k{\mu}_i^2$, where k is a constant [18, 20, 21]. This is becausethe NB distribution accounts for further variance in count outcomes than the Poisson distribution through an additional shape parameter to the Poisson scale parameter [22]. In addition, classical methods of fitting statistical models canbe invalid in the presence of complex sampling designs involvingunequal weights, stratification or multi-stage sampling. To address this concern, there has been a considerable development of methods which do take account of complex designs [23–25]. One advantage of this approach is applicable to a very broad class of complex sampling schemes [26].

Along these lines, in this study researchers fitted a weighted log-linear negative binomial model for the number of modern contraceptive users from service delivery point as the data was over-dispersion due to cluster sampling.

The link function for negative binomial distribution is natural logarithm and then the model can be fitted as: log(*μ*_*i*_) = *Xβ***,** where *μ*_*i*_ be the expected number and variance of women who used the modern contraceptive method in i^th^ SDP, **X** is the predictors and *β* is the parameter of the model. As the data was over-dispersed due to cluster sampling the model leads to the negative binomial with mean and variance of women who used the modern contraceptive method in i^th^ SDP. Here the data was collected from a disproportionate number of population size across nine regions grouped into two residences (rural and urban). Thus in this study, the weighted log-linear model was used that proposed by Agresti [27]. The advantage of the weighted analysis is that it removes the bias due to the unequal population sizes. Then the weighted log-linear model link function can be fitted as: $$ \log \left(\raisebox{1ex}{${\mu}_{ij}$}\!\left/ \!\raisebox{-1ex}{${W}_{ij}$}\right.\right)= X\beta $$**,** where Wij be the total population size in i^th^ region and j^th^ residence, *μ*_*ij*_ be the expected number of women in i^th^ region and j^th^ residence at a given SDP, **X** is the predictors and *β* is the parameter of the model. This model has an equivalent representation as: log*μ*_*ij*_ − log *W*_*ij*_ = *Xβ***,** where -log*W*_*ij*_ is the adjustment term to the log link of the mean called an offset [27]. As the maximum likelihood estimate is biased, restricted maximum likelihood technique was used for parameter estimation [28].

## Result

### Descriptive statistics

Among the study service delivery points (SDP) 206 (44.7%) modern contraceptive providers were health centers whereas 255 (52.3%) were collectively hospital, health post, health clinic, and pharmacy and retail outlet. Out of 10, service delivery points seven had no community health volunteer (CHV) supports, of which more than half 166 (52.2%) were located in the rural area. Among all family planning (FP) services provided at the study SDPs, the smallest were sterilization 76 (2.7%) for female and 57 (2.0%) for male) followed by IUD 268(9.6%) whereas female condom and beads were null. The majority maternal services that offered by SDPs were antenatal care 406(28.3%) followed by delivery 391 (27.3%). The majority 296 (66.8%) of SDPs were offered FP below 5 days per week (Table 1).

**Table 1:** Frequency distribution for qualitative predictors

Variables			Residence area		Total
			Urban	Rural	
Type of facility	Hospital Center	Count (%)	99(48.1)	107(51.9)	206(44.7)
	Others^b^	Count (%)	121(47.5)	134(52.5)	255(55.3)
Advanced Facility	No	Count (%)	24(96.0)	1(4.00)	25(5.4)
	Yes	Count (%)	196(45)	240(55)	436(94.6)
CHV supports	No	Count (%)	152(47.8)	166(52.2)	318(69.0)
	Yes	Count (%)	44(38.9)	69(61.1)	113(24.5)
Types of FP Provide^a^	female sterilization	Count (%)	47 (61.8)	29(38.2)	76 (2.7)
	male sterilization	Count (%)	39 (68.4)	18(31.6)	57(2.0)
	implants	Count (%)	164(43.9)	210(65.1)	374(13.4)
	IUD	Count (%)	152(56.7)	116(43.3)	268(9.6)
	injectables	Count (%)	206(47.5)	228(52.5)	434(15.6)
	pills	Count (%)	211(48.2)	227(51.8)	438(15.7)
	progestin pills	Count (%)	181(52.5)	164(47.5)	345(12.4)
	male condoms	Count (%)	210(48.5)	223(51.5)	433(15.5)
	female condoms	Count (%)	8(80.0)	2(20.0)	10(0.4)
	EC	Count (%)	202(58.0)	146(42.0)	348(12.5)
	beads	Count (%)	1 (50.0)	1(50.0)	2(0.1)
Type of maternal Services Provide^a^	antenatal services	Count (%)	180 (44.3)	226(55.7)	406(28.3)
	delivery services	Count (%)	164(49.2)	169 (50.8)	333(23.2)
	postnatal services	Count (%)	173(44.2)	218(55.8)	391(27.3)
	post-abortion services	Count (%)	161(53.0)	143(47.0)	304(21.2)
Number of days per week FP offered	Below 5 days	Count (%)	133(44.9)	163(55.1)	296(66.8)
	Above 5 days	Count (%)	80(54.4)	67(45.6)	147(33.2)

Key: ^a^ multiple responses, CHVs (Community health volunteers), FP (Family planning), ^b^ includes Hospital / Polyclinic, Health post, Health clinic, Pharmacy and Retail outlet

**Fig. 2:**
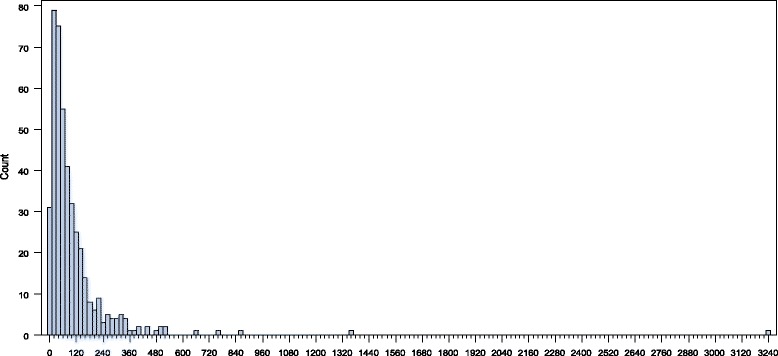
Number of modern contraceptive users

The outcome of interest is the number of modern contraceptive users at SDPs during the last complete month preceding the survey. As a summary of the data, Fig. 2 shows a frequency plot, overall users, in all regions. We observe a highly skewed number of modern contraceptive users, (mean = 105.51 and standard deviation = 195.12), with 1.1% zero values.

Table 2 revealed that the median, quartiles and interquartile range statistic for quantitative variables including the response one. Fifty percent of service delivery points in Ethiopia had given service for 61 modern contraceptive users with the interquartile range of 0.62. Fifty percent of service delivery points in the urban area had given service for 99 modern contraceptive users whereas 50% SDPs in rural had given only to 45 modern contraceptive users. In addition, 50% of service delivery point in urban had 17 nurses whereas 50% SDPs in rural had only 9.

**Table 2:** Descriptive statistic for numerical predictors

	Urban				Rural				Total			
Variables	M	Q1	Q3	IQR	M	Q1	Q3	IQR	M	Q1	Q3	IQR
Number of Modern Contraceptive users	99	45	174	0.59	45	21	74	0.56	61	29	123	0.62
Total number of doctors	0	0	9	1.00	0	0	2	1.00	0	0	5	1.00
Total number of nurses/midwives	17	7	44	0.73	6	0	12	1.00	9	2	28	0.87
Total number of health officers	3	1	6	0.71	2	1	2	0.33	2	1	4	0.60
Total number of pharmacists	2	1	5	0.67	0	0	2	1.00	1	0	3	1.00
Total number of medical assistants	0	0	2	1.00	0	0	0	–	0	0	1	1.00
Total number of other medical staff	4	1	14	0.87	2	1	4	0.60	2	1	8	0.78

Key: M (median), Q1 (lower quartile), Q3 (upper quartile), IQR (inter quartile range)

### Factors associated with the number of modern contraceptive users at SDPs, Ethiopia

The weighted Poisson and a negative binomial regression model were fitted. The deviance values for weighted Poisson and negative binomial regression model were 56821.95 and 351.08 respectively. Thus, the negative binomial regression model estimates the dispersion coefficient as a value 1.52 with a 95% CI 1.30–1.73 signifying that it is more appropriate than the Poisson. Therefore, Table 3 below revealed the regression coefficients, standard errors, the Wald 95% confidence intervals for the coefficients, chi-square tests and *p*-values for each of the model variables based on the analysis of ML parameter estimates.

**Table 3:** Analysis of maximum likelihood parameter estimates

Variable	DF	Estimate	Std Err	95% Wald CI		Chi-Square	Pro>ChiSq	
				Lower	Upper			Exp(Est)
Intercept	1	−3.64	0.60	−4.81	−2.47	37.28	0.00^c^	
Residence of SDP	1					29.79^b^	0.00^c^	
Rural	1	−1.05	0.19	−1.42	−0.68	31.19	0.00^c^	0.35
Urban^a^								
Region of SDP	10					99.43 ^b^	0.00^c^	
Afar	1	4.18	0.73	2.76	5.61	33.14	0.00^c^	65.65
Amhara	1	1.07	0.49	0.11	2.03	4.78	0.03^c^	2.91
Benishangul Gumz	1	0.23	0.65	−1.03	1.50	0.13	0.72	1.26
Dire Dawa	1	0.34	1.01	−1.63	2.31	0.12	0.73	1.41
Gambela	1	−1.64	0.97	−3.54	0.27	2.83	0.09	0.19
Harari	1	−0.02	0.84	−1.68	1.63	0.00	0.98	0.98
Oromiya	1	0.20	0.49	− 0.76	1.15	0.16	0.69	1.22
S.N.N.P	1	−0.30	0.40	−1.09	0.49	0.56	0.45	0.74
Somali	1	−1.55	0.63	−2.78	−0.32	6.08	0.01^c^	0.21
Tigray	1	0.39	0.50	−0.59	1.36	0.60	0.4	1.47
Addis Ababa^a^								
# of days per week FP offered	1					30.86 ^b^	0.00^c^	
Five or below days	1	−1.33	0.26	−1.84	−0.82	26.33	0.00^c^	0.26
Above days^a^								
CHV Supporters	1					0.54 ^b^	0.46	
No	1	−0.20	0.28	−0.74	0.34	0.53	0.47	0.82
Yes^a^								
Type of facility	1					4.80 ^b^	0.03^c^	
Others	1	−0.58	0.26	−1.08	−0.08	5.13	0.02^c^	0.56
Health Center^a^								
Total # of doctors	1	−0.01	0.02	−0.04	0.03	0.32	0.57	0.99
Total # of nurses/midwives	1	0.01	0.00	0.00	0.01	4.21	0.04^c^	1.01
Total # of health officers	1	0.04	0.04	−0.04	0.11	0.87	0.35	1.04
Total # of pharmacists	1	−0.16	0.03	−0.22	−0.10	29.18	0.00^c^	0.85
Total # of medical assistants	1	−0.19	0.04	−0.27	−0.10	19.46	0.00^c^	0.83
Dispersion	1	1.52	0.11	1.30	1.73			

Key: *CI* (Confidence Interval), ^a^ (Reference category), ^b^ (Type 3 chi-square value), ^c^ (the relationship is significant at alpha value of 0.05 and or below), Exp(Est) (Exponentiating estimate)

The variables residence, region, the number of days per week FP offered, type of facility, the total number of nurses/midwives, the total number of pharmacists and the total number of medical assistants were statistically significant. Keeping the other variables constant, the expected log count for a rural modern contraceptive user is 1.05 lower than the expected log count for urban modern contraceptive users. The expected log count of modern contraceptive users at others facility type was 0.58 lower than the expected log count of modern contraceptive users at the health center. The numbers of nurses/midwives were positively affecting the number of modern contraceptive users. Thus, the incidence rate of modern contraceptive users increased by more than one (1.01) as one additional nurse in the given service delivery point (Table 3).

## Discussion

Fifty percent of service delivery points in Ethiopia had given service for 61 modern contraceptive users with the interquartile range of 0.62. Considering the place of residence, urban modern contraceptive users were higher than that of rural modern contraceptive users. The contributors to this positive association may be the better socioeconomic status of women in urban, easy access to family planning services, cultural disparity compared to rural areas, and the high level of women literacy in urban areas. This result is in line with the study conducted at Afghanistan [29].

At the regional level, the disparity was observed among regions.

The expected log number of modern contraceptive users at Gambella, Harari, SNNP, and Somali was lower than that of Addis Ababa. This result is similar with the study in Ethiopia from EDHS data by Tesfaye. He recommended as efficient distribution of health care facilities offering family planning services in urban and rural residents are required and designed for family planning services targeting on Somali region greatly increase the rate of the number of contraceptive use [30]. This is because geographical variations in the number of modern contraceptive use have been found to be influenced by community-level cultural beliefs like value attached to the child, the presence and quality of reproductive health services, shortage of midwives in most SDPs, remote geographical areas, and the presence of transport routes [29, 31–33]. Whereas the expected log number of modern contraceptive users from Afar, Amhara, Benishangul Gumiz, Oromia, Tigray, Dire Dawa was higher than that of Addis Ababa. This result contradicts with the result done by [4] stated as the modern contraceptive prevalence rate in Ethiopia is varied from 1.4% in Somali to 50.1% in Addis Ababa across regions. This contradicts might be mainly due to confounding variables and slightly under estimation in regional towns. The main courses of under estimation may as regional women feeling shame to take contraceptive methods publically.

An increasing of nurse/midwives health officers, the expected log number of modern contraceptive users was increased by 0.01 and 0.04 respectively. Several studies have confirmed to the key role of nurses/midwives and health officers in providing guidance and effective counseling, resulting in an increased number of modern contraceptive users [34, 35]. Other studies have reported an increase in a couple–year protection following the engagement of midwives in family planning services in service delivery points [34, 35]. In Ethiopia, the involvement of health extension workers increased the contraceptive prevalence rate from 14 to 30% in 4 years [34, 35]. In Iran, increased community participation consequent to the involvement of midwives and other stakeholders resulted in the number of modern contraceptive users this intern a decline in total fertility rate. In addition, the incident rate of the modern contraceptive user for below 5 days per week FP offered is 0.35 times compared to above 5 days per week FP offered. This is the fact that increasing the access days to offer modern contraceptive methods leads to increases the number of modern contraceptive users at SDPs.

## Conclusion

This study was aimed to investigate the major factors that affect the number of modern contraceptive users at service delivery point in Ethiopia. Among different factors considered in this study, residence, region, facility type, the number of days per week family planning offered, number of nurses/midwives and number of medical assistants were found to be significantly associated factors for the number of modern contraceptive users in Ethiopia. The influence of these factors can be used to develop the strategies of increasing the number of modern contraceptive users at service delivery points in Ethiopia. The median number of experts at the rural area is very few compared to the urban area in Ethiopia. This intern leads the median number of modern contraceptive users at rural service delivery points in Ethiopia is very few. Few numbers of modern contraceptive users at service delivery points in Ethiopia might potentially lead to high total fertility rate which intern affects the maternal and child health status. Finally, this affects negatively the 2030 ambitious goals for universal access to sexual and reproductive health services, including family planning. Thus, the regional and federal Government of Ethiopia would take immediate steps to address causes of the number of modern contraceptive users in Ethiopia, especially in rural areas.

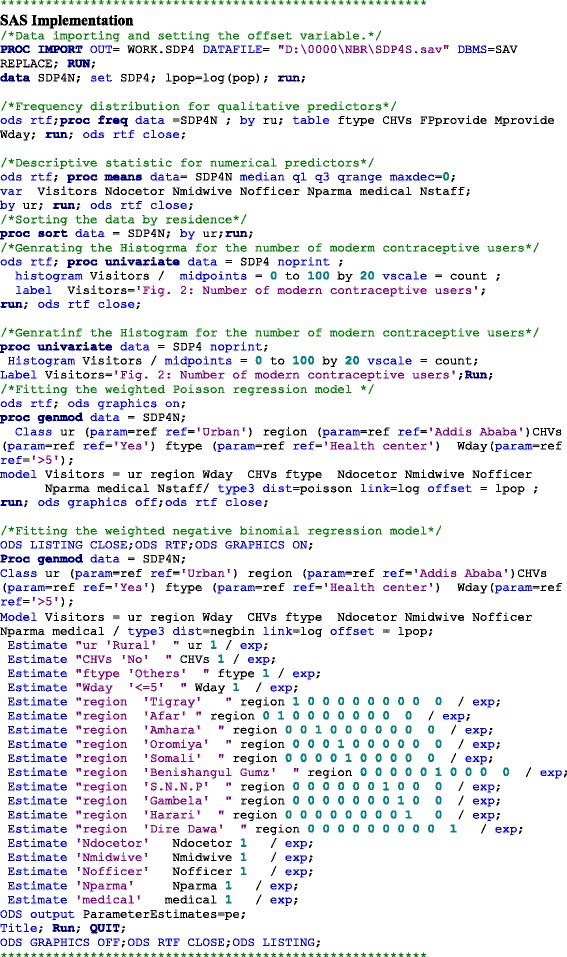



## Abbreviations

AAU: Addis Ababa University
AOR: Adjusted odds ratio
CHS: College of Health Sciences
CI: Confidence interval
COR: Crude odds ratio
EDHS: Ethiopia demographic health survey
ESA: Ethiopian statistical agency
FP: Family planning
GTP: Growth and transformation plan
LAM: Lactational amenorrhea method
OR: Odds ratio
PMA2020: Performance monitoring and accountability 2020
REs: Resident enumerators
SAS: Statistical analysis system
SPH: School of public health
SPSS: Statistical packages for social Sciences
